# ITCH E3 ligase in ATM network

**DOI:** 10.18632/oncoscience.50

**Published:** 2014-06-04

**Authors:** Venturina Stagni, Simonetta Santini, Daniela Barilà

**Affiliations:** Department of Biology, University of Rome “Tor Vergata”, Rome, Italy and Laboratory of Cell Signaling, Istituto di Ricovero e Cura a Carattere Scientifico (IRCCS) Fondazione Santa Lucia, Rome, Italy

Ataxia Telangiectasia Mutated (ATM) kinase is a central regulator of the DNA damage response and its loss of function leads to the development of Ataxia Telangiectasia, a rare genetic disorder characterized by several features among which cerebellar neuron degeneration which causes ataxia, telangiectasia, immune system abnormalities, higher predisposition to lymphoma and leukaemia development and type 2 diabetes. ATM may exert its function at least in part through its kinase activity which is induced in response to a variety of stimuli in addition to DNA damage including, oxidative stress, hypoxia, growth factor stimulation, and in turn triggers the phosphorylation and the modulation of the activity and function of several target proteins[1]. The large number of ATM substrates identified so far, along with the participation of ATM to multiple signaling networks, may contribute to justify the complexity of the A-T phenotype, although the molecular mechanisms underneath have not been fully elucidated yet. Recently ATM kinase has been identified as a novel positive modulator of ITCH E3-ubiquitin ligase activity[2]. ITCH ubiquitinates and controls the protein stability of several substrates therefore impinging on the regulation of a variety of cellular responses, including the DNA damage response, TNFα, NOTCH and HEDGEHOG signaling, the HIPPO pathway and T cell development. Several studies have identified phosphorylation as a major post-translational modification that may promote or repress ITCH activity (reviewed in[3]). Mutagenesis studies have shown that ATM activation in response to DNA damage triggers ITCH phosphorylation on S161. We suggest that phosphorylation on S161 may interfere with the auto-inhibitory intramolecular interaction that locks the HECT domain into an inactive conformation therefore enhancing ITCH activity[2]. The biological significance of ITCH as a novel player of ATM signaling deserves further investigation. One important open issue is whether ATM may impinge on the ubiquitination and degradation of ITCH targets. We could show that in response to DNA damage ATM promotes transiently ITCH enzymatic activity towards two of its well-known substrates, c-FLIP and c-JUN[2]. However, we could also provide evidence that ATM does not promote ITCH-dependent p73 ubiquitination[2], suggesting that the ATM-dependent activation of ITCH may not result in the enhancement of ubiquitination and degradation of the whole set of ITCH substrates. It is plausible that ATM may redirect ITCH on a specific subset of its target proteins. An additional issue arises from the observation that while ITCH directly promotes FLIP and c-JUN ubiquitination, it may require the interaction with additional cofactors to be able to modulate other targets. As an example, GLI-1, a major player of Hedgehog signaling, clearly involved in cerebellum development is regulated by the ITCH-NUMB complex[4]. Therefore, it will be challenging to clarify whether ATM-dependent phosphorylation may impinge on the ITCH-NUMB complex as well. The identification of the role of ATM on ITCH signaling may be relevant as it is tempting to speculate that the alteration of ITCH activity regulation due to the absence of ATM kinase activity may contribute to the Ataxia Telangiectasia pathogenesis. Some answers may be provided by the comparison between ATM deficient mice and ITCH deficient mice. We have shown that Atm−/− mice are resistant to ConA-induced c-FLIP-L downregulation and to ConA-induced hepatocyte cell death[2], similarly to what reported for Itch −/− mice [5] suggesting a role of the ATM/ITCH axis in the modulation of the interplay between death receptors and DNA damage response [2].

The most severe phenotype linked to A-T is cerebellar neurodegeneration. The work of several labs supports the idea that this may be ascribed to the defective response to DNA damage, to ROS accumulation as well as to abnormalities in the control of adult neuronal stem differentiation during adult development [6]. Evidence for a role of ATM in the control of adult neuronal stem cells has been provided suggesting that ATM is required for normal cell fate determination and neuronal survival[7]. The list of ITCH substrates includes proteins involved in stem cell maintenance, such as NOTCH and GLI-1(reviewed in[3]). In particular since GLI-1 has a well established role in HEDGEHOG signaling and in cerebellum development, it will be intriguing to clarify whether ATM may modulate the levels of GLI-1 through the ITCH / NUMB protein complex and whether the loss of adult neuronal stem cell survival and the cerebellar abnormalities described in A-T may be linked to this novel signaling pathway.

**Figure 1:**
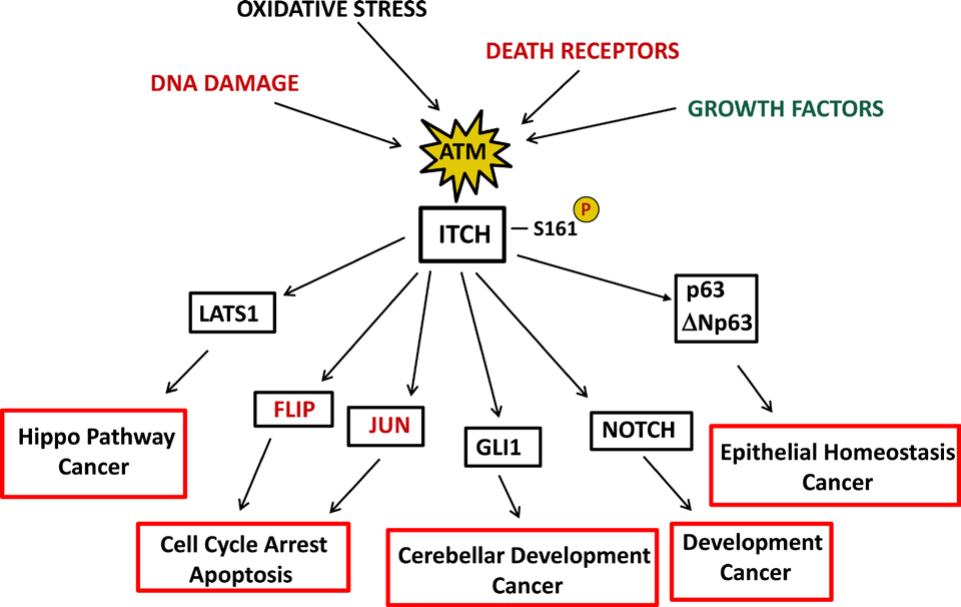
ATM-ITCH signaling may be modulated by several stimuli and its dysfunction may contribute to the complexity of A-T phenotype ATM activation in response to several stimuli may modulate ITCH activity, which in turn may impinge on the stability of some of its substrates. The pathways that may activate the ATM-ITCH axis and its downstream targets are depicted in red, whereas in black are indicated those stimuli and those targets that have not been ascertained yet as novel activators and effectors of this pathway. In the red boxes are indicated the hypothetical biological impact of the ATM-ITCH connection, that may possibly contribute to the A-T phenotype

Future studies will clarify whether ATM may modulate ITCH activity in response to other stimuli in addition to DNA damage and Death Receptors (Figure 1). One additional interesting issue comes from the observation that the modulation of ATM expression and activity by different stimuli may impinge on tumorigenesis and cancer therapy depending on the specific context[8]. As ITCH may modulate several proteins clearly involved in cancer initiation, progression and therapy [3] (Figure 1), it will be intriguing to evaluate whether the ATM-ITCH axis may be relevant in cancer as well.
